# Do anti‐Aβ monoclonal antibodies lower brain plaques in Alzheimer patients through microglia activation?

**DOI:** 10.1002/alz.13684

**Published:** 2024-01-15

**Authors:** Nunzio Pomara, Bruno Pietro Imbimbo

**Affiliations:** ^1^ Nathan S Kline Institute for Psychiatric Research Geriatric Psychiatry Division Orangeburg New York USA; ^2^ Research & Development Department Chiesi Farmaceutici Parma Italy

1

Two recent phase 3 clinical trials in individuals with early Alzheimer's disease (AD) showed that gantenerumab (an amyloid‐β [Aβ] monoclonal antibody [mAb]) did not significantly slow clinical decline compared to placebo.^1^ The authors hypothesized that the lack of beneficial effects might have been related to residual brain amyloid burden and the relatively small number (27.5%) of gantenerumab‐treated subjects who achieved amyloid‐negative status. The authors cited preclinical evidence that gantenerumab removes Aβ through increased microglia‐mediated phagocytosis.

The primary function of microglia cells is to phagocytose protein aggregates and neuronal debris, protect synapses, and support neuronal connectivity. During the early phases of AD, activated microglia surround plaques and fibrils and clear Aβ aggregates and dead neurons through phagocytosis. As Aβ deposition increases and persists over time, microglia switch from a ramified homeostatic state to an amoeboid phenotype (disease‐associated microglia, DAM). With the appearance of tau pathology, the DAM state or microglial activation becomes proinflammatory and associated with greater pathological severity. Eventually, this leads to synaptic damage and neuronal death.^2^

Triggering receptor expressed on myeloid cells 2 (TREM2) is a cell surface receptor of microglia and its stimulation enhances the phagocytic activity of microglia and modulates inflammatory signaling. The activation of TREM2 in response to the presence of Aβ plaques and tau tangles plays a crucial role in the pathogenesis of AD.^3^ A soluble form of TREM2 (sTREM2) promotes microglial activation with stimulation of phagocytic activity and neuronal survival. sTREM2 may exert stage‐specific proinflammatory or anti‐inflammatory effects. Loss‐of‐function *TREM2* gene variants increase the risk of developing AD by impairing the beneficial microglia activation. Some *TREM2* mutations have been associated with reduced cerebrospinal fluid (CSF) sTREM2 levels consistent with decreased TREM2 expression/engagement. Different studies have shown that in both mild‐to‐moderate and early AD the CSF sTREM2 concentrations are higher compared to cognitively unimpaired controls. In individuals with early AD, increased CSF sTREM2 levels indicate a change in microglial activation. As the disease progresses, CSF sTREM2 exhibits a dynamic response and may serve as a valuable biomarker in clinical trials. Decreased levels of CSF sTREM2 are linked to Aβ pathology^4^ while increased levels are associated with tau‐related neurodegeneration.^5^ A 3‐year study of 231 AD patients showed that higher sTREM2 levels at baseline were linked to slower clinical progression.^6^ A 3‐year study of 148 presymptomatic autosomal‐dominant AD subjects indicated that higher annual rates of increase in sTREM2 corresponded to a reduced rate of cognitive decline.^7^

Anti‐Aβ mAbs work through multiple mechanisms to clear Aβ.^8^ In the blood, these drugs bind to Aβ preventing it from entering the brain across the blood‐brain barrier. The modification of the equilibrium between monomeric Aβ in the brain and blood facilitates the efflux of Aβ from the central nervous system to the CSF and the periphery. A low proportion (0.1% to 0.3%) of anti‐Aβ mAbs enters the brain and binds directly to Aβ aggregates. Some antibodies can solubilize Aβ protofibrils and inhibit their aggregation into fibrils and plaques. Importantly, monoclonal anti‐Aβ antibodies can opsonize Aβ aggregates marking them for recognition by microglia. Subsequently, microglia phagocytose and degrade Aβ aggregates, effectively clearing Aβ from the brain.

There are few data available on the effects of anti‐Aβ mAbs on sTREM2 levels in the CSF of patients with AD. Data are only available for crenezumab and gantenerumab. There are no published data on lecanemab, donanemab, or aducanumab.

A 105‐week study on crenezumab (CREAD) in individuals with early AD reported the effects of the drug on CSF sTREM2 levels. The study was discontinued following a preplanned interim analysis indicating that the CREAD study was unlikely to meet the primary end point.^9^ At week 105, CSF sTREM2 data were available for 24 patients treated with placebo and 18 with crenezumab. Compared to baseline, there was a slight mean increase in sTREM2 levels in the placebo group (+0.45 ± 0.32 ng/mL, +4.3%), while mean sTREM2 levels remained stable (−0.09 ± 0.38 ng/mL, −0.8%) in the crenezumab group. The difference between the two treatment groups was not statistically significant. No meaningful changes in other AD biomarkers were observed in this study, including a very marginal effect on Aβ‐PET (−2 Centiloids vs placebo). At week 105, the drug did not improve the clinical performance of the patients compared to placebo as assessed with the Clinical Dementia Rating Scale‐Sum of Boxes (CDR‐SB; mean difference of −0.17 points in favor of placebo).

Available data on the effects of gantenerumab on CSF sTREM2 levels derives from two 116‐week studies of early AD (GRADUATE I and II studies).^1^ Data at week 116 are available only for 12 patients on placebo and 12 on gantenerumab.^10^ Compared to baseline, sTREM2 levels slightly increased by 7.4% in the pooled placebo group, while in the pooled gantenerumab group, mean levels remained stable (−1.5%). In the two GRADUATE studies, gantenerumab treatment significantly decreased amyloid burden compared to placebo (−66.4 and −56.5 Centiloids, respectively), although the effect of the drug on CDR‐SB was not statistically significant (−0.31 and −0.19 points, respectively).

Very limited data are available on the effects of anti‐Aβ mAbs on microglial activation in AD individuals. Crenezumab and gantenerumab have virtually no effect on sTREM2, and no significant effects on cognition. There are no published data on lecanemab, donanemab, or aducanumab. We believe this is an important piece of missing information especially for those drugs approved by the FDA. It is commonly believed that the cognitive and clinical response to anti‐Aβ mAbs is linked to the extent and rate of removal of amyloid from the brain. It is not fully understood why some anti‐Aβ mAbs are more efficient than others in removing brain amyloid. Preclinical studies have hypothesized that the mechanism of brain removal by anti‐Aβ mAbs is mainly due to microglial activation. We believe that it would be useful to determine the effects of approved anti‐AD drugs on CSF levels of sTREM2 to know if these effects contribute to brain plaque removal. In the presence of brain tau pathology, higher CSF sTREM2 has been associated with increased neuroinflammatory and Aβ deposition. Thus, examining the effects of anti‐Aβ mAbs on CSF sTREM2 may be important also in later stages of the disease.

## CONFLICT OF INTEREST STATEMENT

Dr. Nunzio Pomara has served as Investigator in several anti‐Alzheimer's and anti‐depressant drug trials. He holds a joint patent with NYU on potential Aβ disturbances in depression. Dr. Bruno P. Imbimbo is an employee at Chiesi Farmaceutici. He is listed as an inventor in a number of Chiesi Farmaceutici's patents of anti‐Alzheimer drugs. Author disclosures are available in the supporting information.

## Supporting information

Supporting Information
